# Curcumin-Loaded Nanoparticles with Low-Intensity Focused Ultrasound-Induced Phase Transformation as Tumor-Targeted and pH-Sensitive Theranostic Nanoplatform of Ovarian Cancer

**DOI:** 10.1186/s11671-020-03302-3

**Published:** 2020-04-07

**Authors:** Xiaoxia Guo, Jie Mei, Yong Jing, Shiguang Wang

**Affiliations:** 1 Department of Obstetrics and Gynecology, Sichuan Academy of Medical Sciences & Sichuan Provincial People’s Hospital, Chengdu, 610041 Sichuan China; 2 Department of Imaging, Eastern Hospital of Sichuan Academy of Medical Sciences & Sichuan Provincial People’s Hospital, No. 585 Honghe North Road, Longquanyi District, Chengdu, 610000 Sichuan China

**Keywords:** Low-intensity focused ultrasound, Ferritin, Theranostic, Curcumin, Perfluorohexane

## Abstract

We have developed a simple and versatile nanoplatform using pH-sensitive ferritin nanocages co-loaded with the anticancer drug curcumin (Cur) and liquid fluorocarbon perfluorohexane (PFH) inside the core and conjugated tumor-targeting molecule FA outside the shell referred to as FA-FCP. The synthesized FA-FCP has an average particle diameter of 47 nm, with stable and favorable physicochemical properties in different media, and high biocompatibility and biosafety in vivo and in vitro. Under the conditions of low-intensity focused ultrasound (LIFU) and at pH = 5.0, FA-FCP released a large amount of drugs (53.2%) in 24 h. After 4 min of LIFU (7 W) treatment, FA-FCP provided contrast-enhanced ultrasound imaging capabilities at pH = 5.0. Due to FA receptor-mediated endocytosis, FA-FCP could efficiently enter the cells and further relocate to lysosomes. Eighteen hours after injection of FA-FCP, the tumor was stimulated by LIFU, resulting in a contrast-enhanced ultrasound image. In vivo and in vitro experiments showed that the combined use of FA-FCP and LIFU had significant tumor treatment effects. Based on the results, it was concluded that FA-FCP combined with the external LIFU and the endogenic acidic environment can have powerful theranostic functions and provide a novel type of non-invasive and integrated tumor theranostic option.

## Introduction

Ovarian cancer is a highly metastatic and lethal disease with a high mortality rate [1, 2]. As the early clinical symptoms are not conspicuous, tumor cells have already become highly metastatic when most patients are diagnosed [3]. The commonly used treatments in the clinic are combined cytoreductive surgery and chemotherapy, and the 5-year survival rate of patients with advanced disease is very low [4]. Therefore, it is urgent to develop new strategies for ovarian cancer treatment and diagnosis to improve the overall survival of patients. Recently, a nanoplatform that combines targeted therapy and imaging diagnostic functions has provided an alternative treatment strategy for the effective treatment of tumors [5–7].

In recent years, acoustic-response nanoplatform (ARN) has widely been used in tumor treatment and diagnostic research [8, 9]. ARN is usually designed as microbubbles (MBs) or nanoparticle (NPs) models [10–12]. Compared with MBs, NPs have a smaller particle size, higher permeability, longer pharmacokinetic cycle, better stability, and easier surface modification ability [13]. Different kinds of phase-transformable liquid fluorocarbons, such as perfluoropentane (PFP), perfluorohexane (PFH), and perfluorooctyl bromide (PFOB), are commonly used to achieve acoustic responsiveness [14–19]. These liquid fluorocarbons could produce bubbles, or even bursts, via acoustic droplet vaporization (ADV) effects under external focused ultrasound stimulation. This process provides the ARN with enhanced ultrasound contrast capabilities. Among these liquid fluorocarbons, PFP has a low boiling point (29.2 °C) and is prone to gasification in the body, leading to gas embolism; PFOB has a very high boiling point (144 °C) and requires ultra-high-intensity ultrasound to trigger liquid-vapor phase changes. Therefore, PFH is considered an ideal phase-transformation material with a boiling point of 56 °C. However, PFH is hydrophobic, and it is a good idea to encapsulate it inside the nanoparticles to make it water-soluble. At present, various materials, such as liposomes, PLGA, organic mesoporous, organic polymer, etc. have been reported for encapsulating liquid fluorocarbons [20–24]. At the same time, these carriers can be loaded with anticancer drugs to enable ARN to have chemotherapeutic functions and to show the ability of acoustic-controlled drug release [16, 19]. Although these acoustically responsive nanoplatforms have been reported to exhibit good tumor diagnosis and treatment integration capabilities, the biocompatibility of nanocarriers is still a concern for clinical transformation. In recent years, iron-free ferritin nanocages have been widely used as drug delivery vehicles because they are endogenous proteins and have significant pH sensitivity [25–27]. They can decompose in acidic environments and reassemble in alkaline environments. This makes ferritin highly biocompatible and providing it with controlled drug loading and releasing capacity.

In this study, ferritin was used as a carrier to load both PFH and curcumin (Cur) and modify the tumor-specific targeting molecule FA on the protein surface to obtain a multifunctional nanoplatform (FA-FCP). Curcumin is a natural polyphenol, which is extracted from turmeric and has been reported to have favorable anticancer effects; however, its water solubility is poor [28–31]. FA-FCP improves the water solubility of PFH and Cur, has high physiological stability in different media, and has favorable biocompatibility and biosafety in vivo and in vitro. Under the conditions of LIFU and at pH = 5.0, FA-FCP releases a large amount of drugs in 24 h (53.2%). After 4 min of LIFU (7 W) treatment, FA-FCP at pH = 5.0 provides contrast-enhanced ultrasound imaging. Due to FA receptor-mediated endocytosis, FA-FCP can easily enter cells and relocate in lysosomes. Eighteen hours after the injection of FA-FCP, the tumor site was stimulated by LIFU, and the tumor showed contrast-enhanced ultrasound imaging. In vivo and in vitro experiments have shown that FA-FCP has a significant effect on tumor treatment. These results demonstrated that the advantage of non-invasive, ligand/receptor-mediated targeting, LIFU-triggered phase transition, or even blasting, and precise drug release make FA-FCP a promising tumor theranostic nanoplatform.

## Materials and Methods

### Materials

Ferritin (FRT), folic acid (FA), and perfluorohexane (PFH) were purchased from Sigma (St. Louis, MO, USA). Curcumin (Cur) was purchased from Aladdin Industrial Corporation (Shanghai, China). NH_2_-PEG_2000_-FA and NH_2_-PEG_2000_-COOH were supplied by Xi’an Ruixi Biotechnology Co., Ltd (China). 1-ethyl-3-(3-dimethylaminopropyl) carbodiimide (EDC) and *N*-hydroxysuccinimide (NHS) were bought from Thermo Fisher Scientific (Waltham, MA, USA). Cell Counting Kit-8 (CCK-8) was obtained from Dojindo Laboratories (Kumamoto, Japan). Dulbecco’s modified Eagle’s medium (DMEM), phosphate-buffered saline (PBS), penicillin-streptomycin, trypsin-EDTA, and fetal bovine serum (FBS) were purchased from Gibco (Grand Island, NY, USA).

### Preparation of FA-FCP

In order to prepare FA-FCP, firstly, 10 mg FRT was dissolved in 10 ml water. A total of 10 mg Cur was dissolved in 0.3 ml DMSO. The two kinds of solutions and 1 ml PFH were mixed under pH 5.0 condition and 30-min ice-bath ultrasonication. After that, the pH value of the mixture was adjusted to 7.4 to obtain Cur and PFH loaded FRT (FCP). Secondly, target molecule FA was covalently conjugated with FCP through carbodiimide method [8]. In brief, 5 mg NH_2_-PEG_2000_-FA was added into the FCP solution above with the presence of EDC (5 mg/ml) and NHS (20 mg/ml). After 3-h reaction at room temperature with slight stirring, the mixture was purified through dialysis against distilled water (MW cut off = 12 kDa) for 24 h, resulting FA-conjugated FCP (FA-FCP). The Cur loading ratio was detected by a UV–Vis spectrophotometer at 426 nm and calculated as (A_a_−A_b_)/A_c_, where A_a_, A_b_ and A_c_ represent the weight of the FA-FCP, FA-FP, and FA-FP, respectively.

### Characterizations

The sizes and zeta potentials of the nanoparticles were tested by a Zeta Sizer (Malvern, NanoZS, UK). The morphology of the nanoparticles was observed by transmission electronic microscopy (TEM, Hitachi, Japan) and atom force microscopy (AFM, Agilent Technologies 5500LS, Chandler, Arizona). The cellular uptake of the nanoparticles was detected by confocal laser scanning microscopy (LEXT OLS4100, Olympus, Japan) and flow cytometry (BD, Franklin Lakes, NJ). Absorption spectra were acquired by a UV–Vis spectrophotometer (UV1800, Shimadzu, Japan).

### Cell Culture and Animal Model

Human ovarian cancer cell line SK-OV-3 was provided by Shanghai Institute of Cell Biology, Chinese Academy of Sciences. The cells were cultured in DMEM media containing 10% fetal bovine serum and 1% penicillin-streptomycin solution in 5% CO_2_ at 37 °C.

Balb/c nude mice (female, about 5 weeks) were provided by Vital River Laboratory Animal Technology Co., Ltd. (Beijing, China). For SK-OV-3 tumor model, 1 × 10^6^ cells were injected into the right back region of mice subcutaneously. All experimental procedures were performed in accordance with the guidelines for the Care and Use of Laboratory Animals of Sichuan Academy of Medical Sciences and were approved by the Ethics Committee of Sichuan Academy of Medical Sciences.

### pH/LIFU Triggered Cur Release

The FA-FCP aqueous solution was divided into four portions and loaded into a dialysis bag (MW 5000). They were then dialyzed into 10 ml of PBS solution having a pH of 5.0 and 7.4, respectively. After 3 h, the aqueous solution was treated with or without LIFU (7 W, 5 min) (50% duty cycle, pulse wave mode and the following procedure kept consistent). At a specific time point, 1 ml of dialysate was removed and an equal volume and equal pH blank solution was added. The dialysate was taken out and measured by a UV–Vis spectrometer, and the concentration of Cur was calculated.

### ADV Properties and US Imaging Function of FA-FCP

FA-FCP in pH = 5.0 and 7.4 condition were irradiated with different power of LIFU (5, 6, 7 W) and for different total of time (3, 4, and 5 min) in the agarose gel model, then US images were observed by US equipment (Esaote Mylab 90, Italy) with the frequency of 5–12 MHz and mechanical index (MI) of 0.06. The US intensity of the region of interest in the US image was analyzed by ImageJ software.

### Targeting Ability of FA-FCP

To demonstrate the targeting ability of FA-FCP in vitro, FITC was used to label the nanoparticles. Briefly, 1 mg of FITC was dissolved in 1 ml of DMSO and then mixed with FCP and FA-FCP for 30 min with gentle agitation. Then, the mixed solution was dialyzed overnight in deionized water to remove free FITC and DMSO to obtain FITC-labeled nanoparticles. The cells were cultured for 24 h, and then the FITC-labeled nanoparticles were added into the culture plates for 3-h incubation. Afterwards, the cells were washed with PBS for three times and then stained with DAPI for 5 min, lysotracker red for 10 min, fixed with 4% paraformaldehyde for 15 min. Finally, the cells were observed by a confocal laser scanning microscope. The statistical fluorescence signals inside cells were measured and analyzed by flow cytometry.

### Blood Circulation and Tumor Accumulation of FA-FCP

Free Cur and FA-FCP (with same concentration of Cur) were intravenously injected into normal mice. And then, the mouse blood in various groups were collected at different time points from the orbital plexus and were dissolved in lysis buffer. The Cur concentration in the blood in these treated groups was determined by Cur absorbance spectra of each solubilized blood sample using an UV–Vis spectrometer and was defined as the percentage of injected dose per gram of tissue (ID%/g).

The content of FA-FCP in tumor was performed in tumor-bearing mice. After 0, 1, 6, 12, 18, and 24 h intravenous injection of the FA-FCP, the tumor tissues were collected, weighed, and digested by aqua regia solution for 24 h. The Cur concentration in the blood in these treated groups was determined by Cur absorbance spectra of each solubilized tumor tissue by an UV–Vis spectrometer and was defined as the percentage of injected dose per gram of tissue (ID%/g).

### In Vitro and In Vivo Anticancer Efficacy

For in vitro anticancer efficacy, the cells were treated PBS, Cur, FA-FCP, FCP + LIFU and FA-FCP + LIFU (at the same Cur dose of 5 mg/kg) for 3 h, and then were irradiated by LIFU (5 min, 7 W). The treated cells were incubated for another 21 h. After that, the viability of the treated cells was detected by CCK-8 assay.

For in vivo anticancer efficacy, tumor-bearing mice were randomly divided into five groups (*n* = 6) and then were intravenously injected with saline (control), free Cur, FA-FCP, FCP + LIFU, and FA-FCP + LIFU, respectively. During 24 days of treatment, the tumor volume and body weight of the mice were monitored every 3 days. The size of the tumor was detected by a caliper. The tumor volume = length×width^2^/2. The tumor growth change was showed by relative tumor volumes which were calculated as V/V_0_, where V_0_ represents the initial tumor volume.

### Biosafety Evaluation

SK-OV-3 cells were seeded in a 96-well plate at the density of 1 × 10^4^ cells/well and allowed to attach for 24 h. Various concentrations (0, 20, 40, 100, 200, and 500 μg/ml) of FA-FRT-PFH were cultured with SK-OV-3 cells. After 24-h treatment, the cells were treated by DMEM media containing 10% CCK-8 for 20 min in an incubator. The absorbance of the cells at 450 nm wavelength was detected by a Multimode Plate Reader (Thermo Scientific) to characterize the cell viability. Furthermore, the major organs including the heart, liver, spleen, lung, and kidney of healthy mice at 25 days post-injection of FA-FCP were collected and analyzed by hematoxylin and eosin staining. The HE staining images were observed using an optical microscopy.

### Statistical Analysis

All data were presented as mean ± standard deviation. The statistical data were processed with SPSS 22.0 software. The Student’s *t* test was performed to determine the statistical significance between the two groups. *P* < 0.05 represents a significant difference.

## Results and Discussion

### Preparation and Characterizations of FA-FCP

**Scheme 1:**
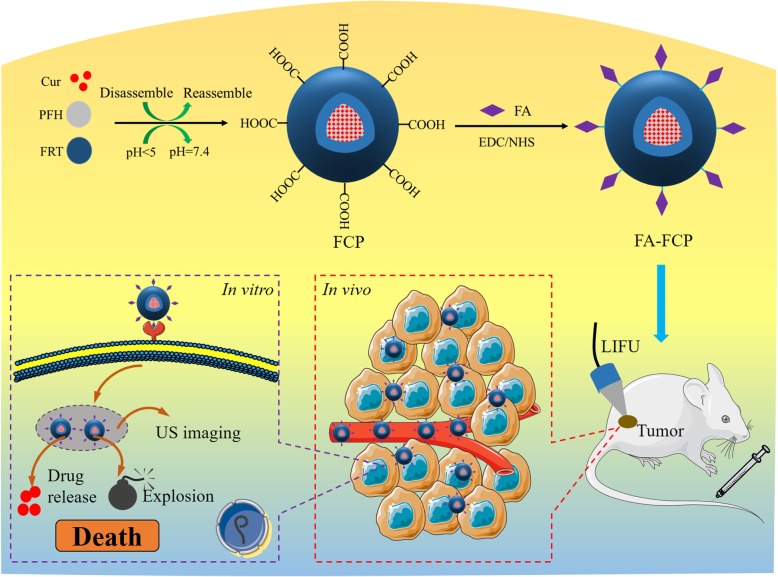
Schematic representation of the assembly method, synthesizing process and theranostic application of the FA-FCP.

**Fig. 1:**
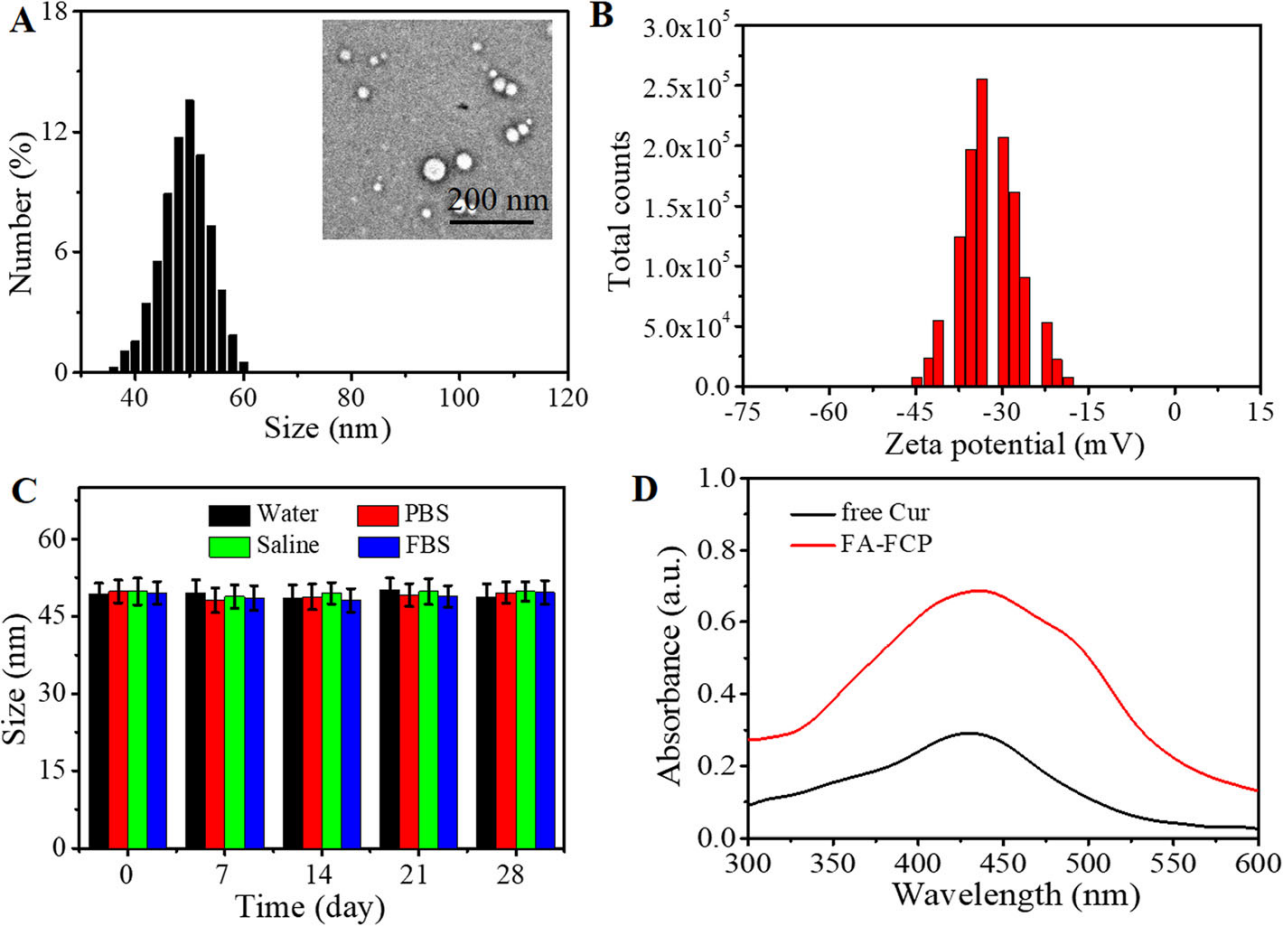
The characterization of FA-FCP. **a** Size distribution of FA-FCP. Inset is the TEM image of FA-FCP. **b** Zeta potential of FA-FCP. **c** Size change of FA-FCP in water, phosphate-buffer saline (PBS), saline and fetal bovine serum (FBS) in 28 days. **d** The UV–Vis-NIR absorption curve of free Cur (50 μg/ml) and FA-FCP (40 μg/ml).

Scheme 1 illustrates the synthesis of FA-FCP and its application in tumor ultrasound (US) imaging and combinational US chemotherapy in vitro and in vivo*.* The multifunctional theranostic agent FA-FCP was prepared through a simple and biocompatible self-assembly method. The TEM and AFM images of FA-FCP show a spherical structure (Fig. 1a, inset and Additional file 1: Figure S1). DLS analysis showed that FA-FCP was approximately 47 nm in average diameter and −37 mV in average zeta potential in water (Fig. 1a and b). After 4 weeks in water, phosphate-buffered saline (PBS), saline, and FBS, FA-FCP displayed stability in size (Fig. 1c), indicating that the prepared FA-FCP had favorable physiological stability, likely due to PEG coating and the nature of FRT [32]. The UV–vis–NIR spectrum of FA-FCP displayed the absorption peak of Cur, demonstrating the existence of Cur in FA-FCP. The Cur loading ratio was 125.8 ± 2.1%.

**Fig. 2:**
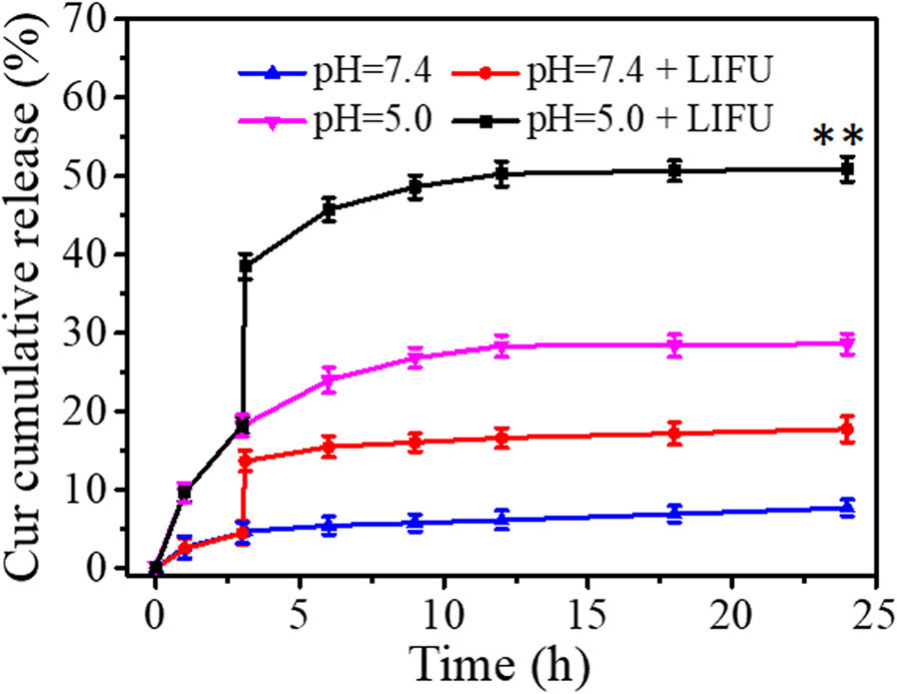
Drug cumulative release of FA-FCP in PBS (pH = 5.0/7.4) at 37 °C with or without LIFU after 3 h. **p < 0.01, compared with the other groups

Figure 2 shows the Cur-release profile from FA-FCP under different pH conditions with or without LIFU. Without LIFU irradiation, Cur released at pH = 5.0 was about 30% over 24 h, which was significantly higher than that at pH = 7.4 (5.3%). However, under LIFU (7 W, 4 min), Cur released at both pH = 5.0 and pH = 7.4 conditions showed a sharp increase. However, compared with the Cur released at pH = 7.4, the Cur released at pH = 5.0 (50%) was obviously higher over 24 h. This feature makes FA-FCP very useful in the tumor microenvironment. The excellent drug release is attributed to (1) FRT under acid condition could disassemble and open the nanocage to release the loaded Cur; (2) LIFU induced the instantaneous phase transition that allowed Cur to escape from the expanded porous shell steadily.

### In Vitro ADV Effect of FA-FCP

**Fig. 3:**
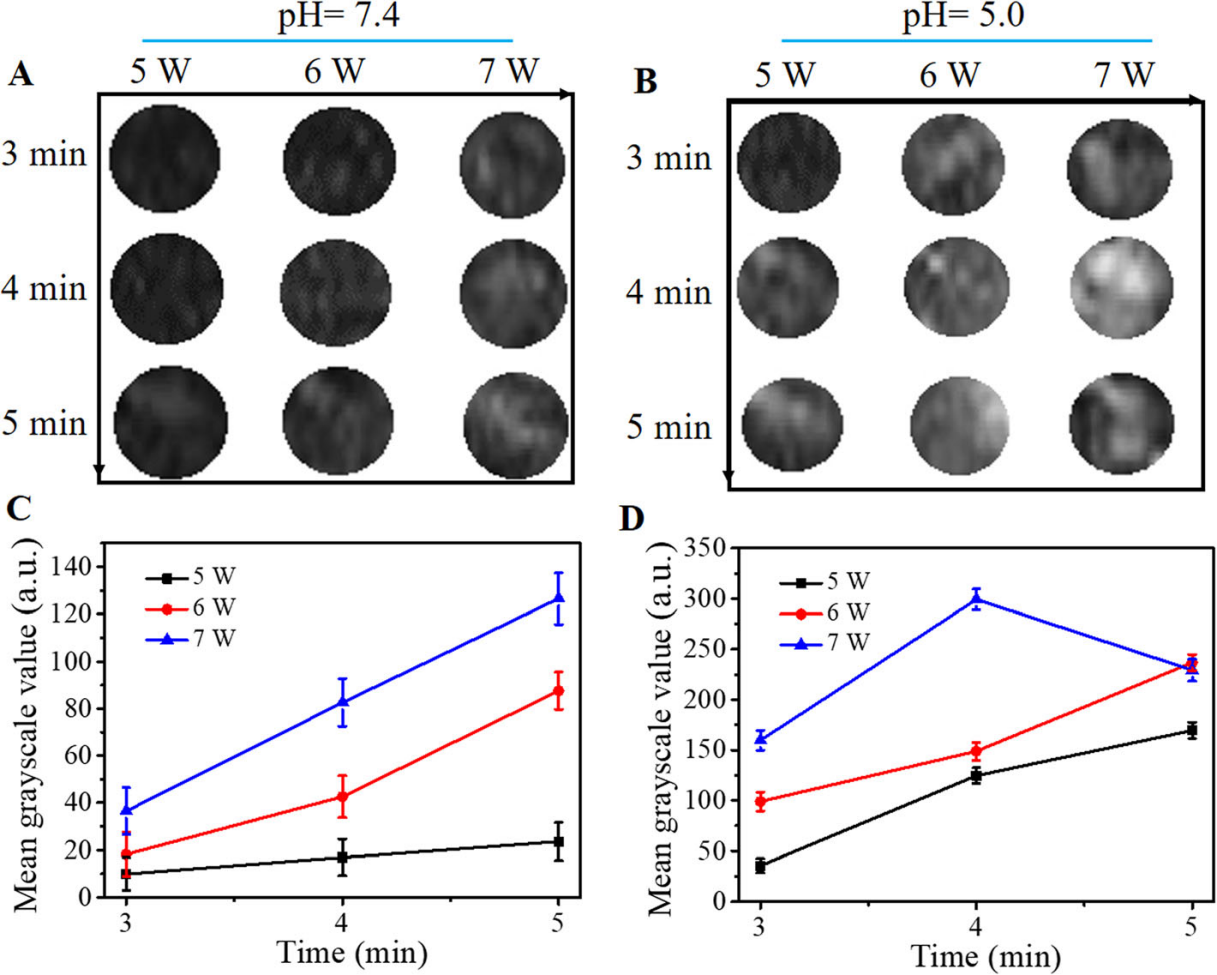
**a** and **b** US images of FA-FCP mixed with agarose gel at different duration and power of LIFU at pH 5.0/7.4. **c** and **d** The corresponding statistical data of US signal in Fig. 3a and b

The FA-FCP was exposed to LIFU with a power of 5, 6, and 7 W, respectively, and a duration of 3–5 min at pH = 7.4 or 5.0 to evaluate the best condition of power and duration of LIFU and pH value. The US intensity represents the ADV effect. According to US images (Fig. 3a and b) and average grayscale values in US images (Fig. 3c and d), the ADV effect exhibited a time/power-dependent trend at pH = 7.4, which peaked at a parameter of 7 W for 5 min. However, at pH = 5.0, the ADV effect peaked at a parameter of 7 W for 4 min. When the parameter was lower than 5 W for 3 min, there were insufficient triggered bubbles to optimize US images; however, once the parameter exceeded 7 W for 4 min, most of the generated bubbles collapsed and gradually disappeared. The results above indicated that a certain stimulation was essential to arouse the ADV effect of FA-FCP, which was a 7-W power and a 4-min duration at pH = 5.0.

### The Targeting Ability of FA-FCP

**Fig. 4:**
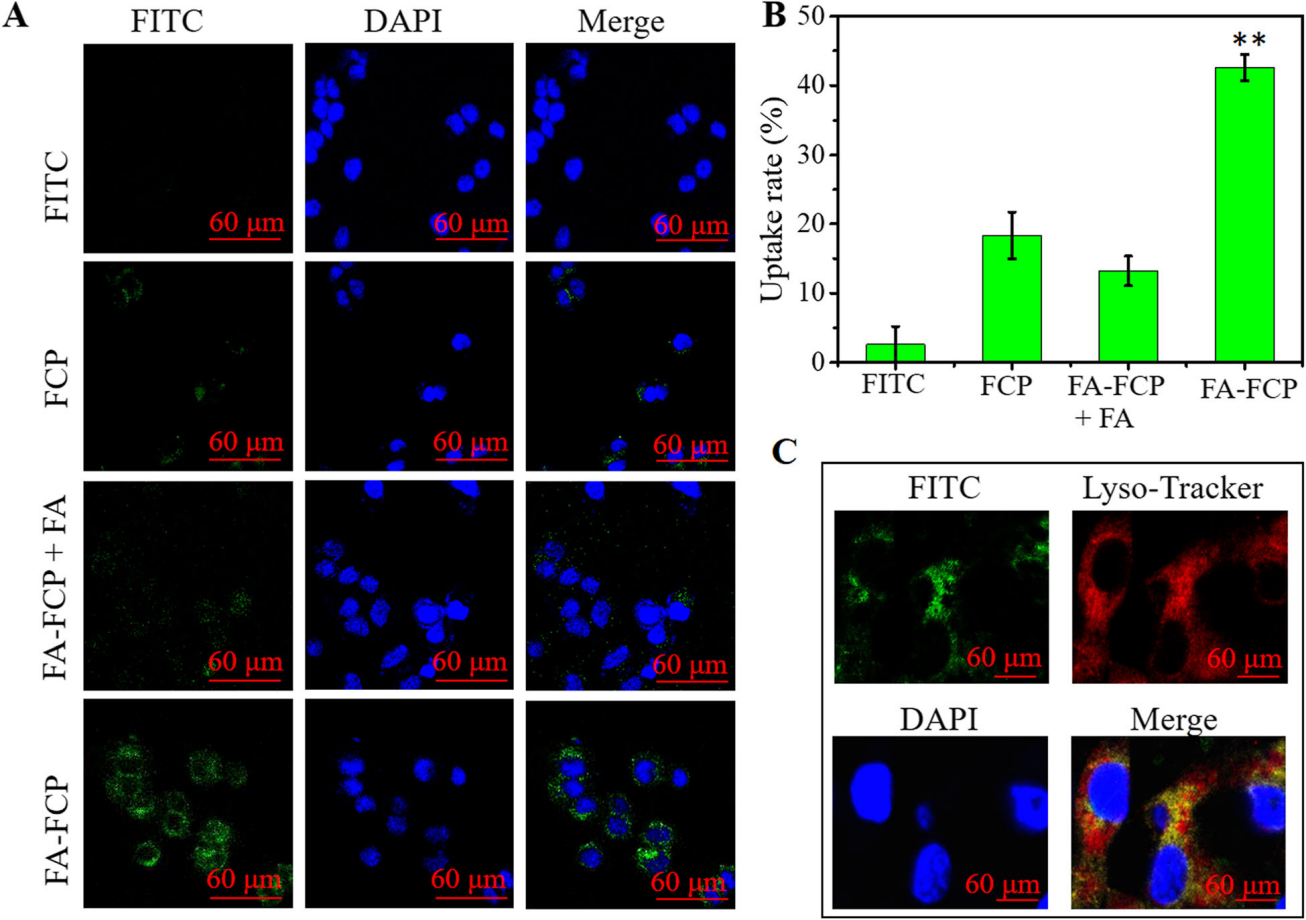
**a** The confocal fluorescence images of cells treated with free FITC and FITC labeled FCP, FA-FCP + FA and FA-FCP. Green and blue colors represented FITC and DAPI fluorescence, respectively. Scale bar = 60 um. **b** The FITC fluorescence signal statistical data inside cells treated with free FITC and FITC-labeled FCP, FA-FCP + FA and FA-FCP by FCM. **p < 0.01, compared with the other groups. **c** The confocal fluorescence images of cells treated with FITC-labeled FA-FCP and lyso-tracker red. Green, blue and yellow colors represented FITC, DAPI, and green/blue merged fluorescence, respectively. Scale bar = 60 um

A classic small-molecule dye, FITC, was used to label the nanoparticles. As shown in Additional file 1: Figure S2, the fluorescence intensity of FITC-labelled FCP and FA-FCP at the same concentration showed no significant difference, indicating that the labelled FITC amount difference on FCP and FA-FCP could be not influent the cell uptake experiment. Fluorescence images and FCM analysis showed that some FCP could enter cells with a 19.5% uptake ratio (Fig. 4a and b), possibly because the cell’s surface has some ferritin receptors that promote the internalization of FCP. After conjugation with FA, FA-FCP showed a high fluorescence signal in cells with 44.3% of uptake ratio, and an uptake ratio collapse (13.8%) when the cells were pretreated with free FA (Fig. 4a and b). The results above indicated that FA conjugation largely increased the uptake ratio of FA-FCP through the FA-receptor-mediated endocytosis effect.

Furthermore, the organelle localization of FA-FCP was investigated using a lysosome-specific staining dye (Lyso-Tracker Red). As shown in Fig. 4c, FA-FCP- and Lyso-Tracker Red-treated cells exhibited strong green and red fluorescence in the cytoplasm, respectively. After the merging of green and red, intense yellow fluorescence was visible in the cytoplasm, indicating that FA-FCP could relocate to lysosomes (pH ≈ 5.0), which was beneficial for triggering the drug release in the cells.

### Blood Circulation and Tumor Accumulation of FA-FCP

**Fig. 5:**
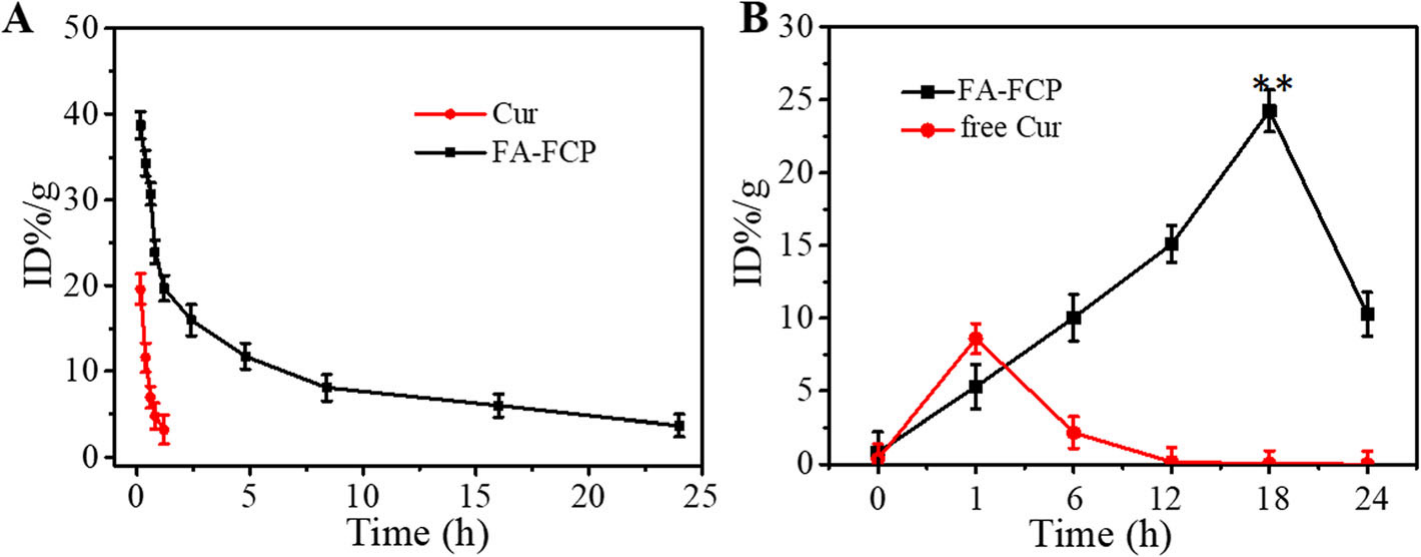
**a** The blood circulation of free Cur and FA-FCP after injection into mice. **b** The content of free Cur and FA-FCP in tumor tissue after injection into tumor-bearing mice. **p < 0.01, compared with the other groups

As shown in Fig. 5a, the half-life of FA-FCP was about 7.31 h (an 8-fold increase), compared with that of free Cur, likely due to the PEG coating and FRT encapsulation. This prolonged half-life of FA-FCP in the bloodstream is beneficial to improving drug retention in the systemic circulation, facilitating the accumulation of drugs at tumor sites [32, 33]. Furthermore, the dynamic accumulation of free Cur and FA-FCP in the tumor from 0 h to 24 h after injection of the nanoparticle is shown in Fig. 5b. As can be seen, FA-FCP showed the highest accumulation of 18 h and free Cur showed the highest accumulation of about 1 h after injection. These results indicate that compared with free Cur, FA-FCP had a significantly higher accumulation performance in the tumor tissue, possibly due to the enhanced permeability and retention (EPR) and the FA receptor-mediated active targeting effect [34, 35].

### In Vivo US Imaging

**Fig. 6:**
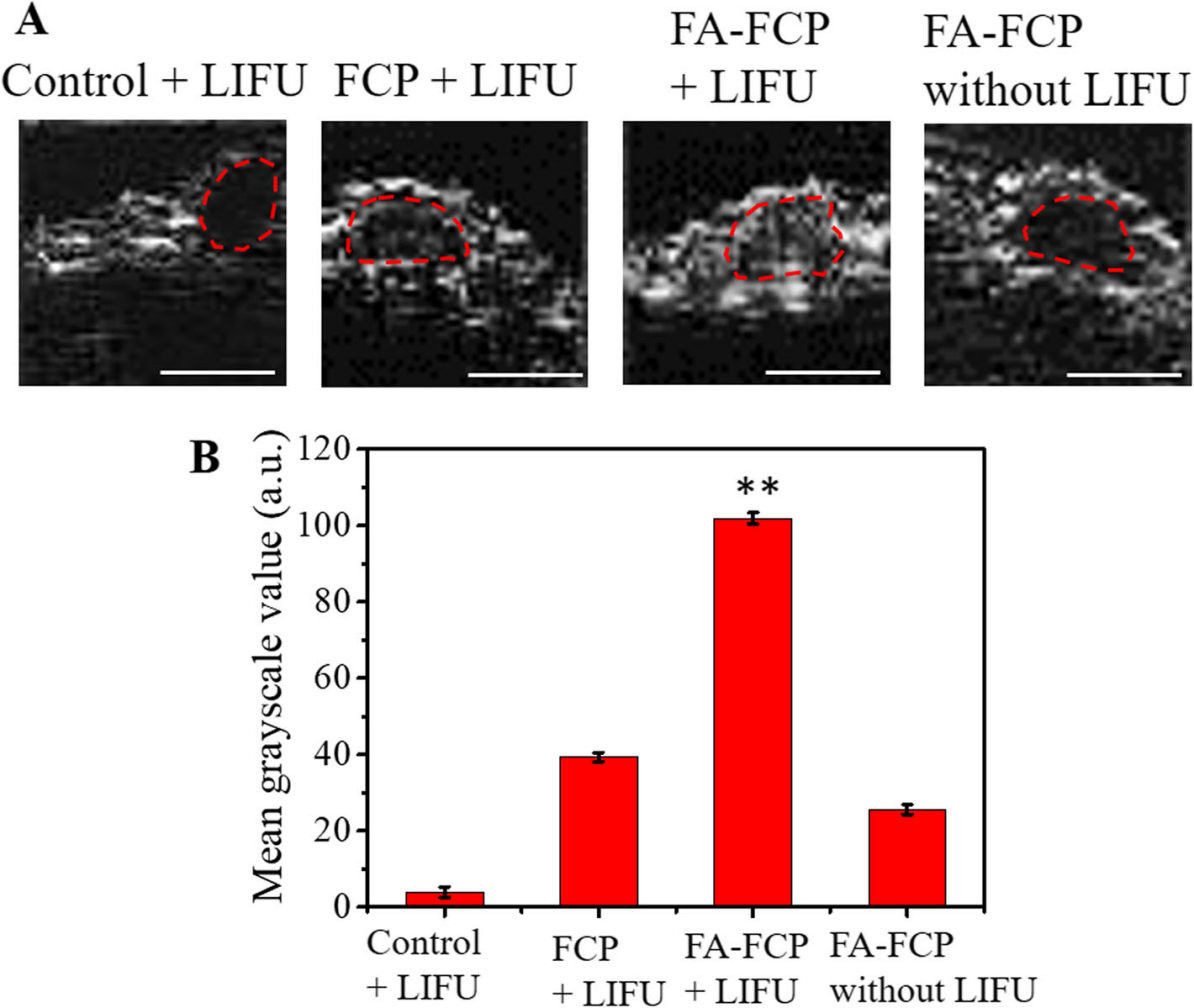
**a** US images of tumor in control + LIFU, FCP + LIFU, FA-FCP + LIFU, and FA-FCP without LIFU groups, respectively. **b** The quantitative mean grayscale values of US images of tumor site in different groups. **p < 0.01, compared with the other groups

The tumor of nude mice treated in four groups (blank control + LIFU, FCP + LIFU, FA-FCP + LIFU, FA-FCP without LIFU) were observed with or without being exposed to LIFU (7 W, 4 min) to further explore the ADV potential of FA-FCP in vivo. As shown in Fig. 6a, no obvious US signal was observed inside the tumor under LIFU irradiation in the control group. At 18 h after injections, a significantly stronger US signal was seen inside the tumor in the FA-FCP + LIFU group compared with the groups with FCP + LIFU, and FA-FCP without LIFU (Fig. 6a and b). The results demonstrated that FA-FCP + LIFU could enhance the US imaging contrast of the tumor.

### In Vitro and In Vivo Anticancer Therapy

**Fig. 7:**
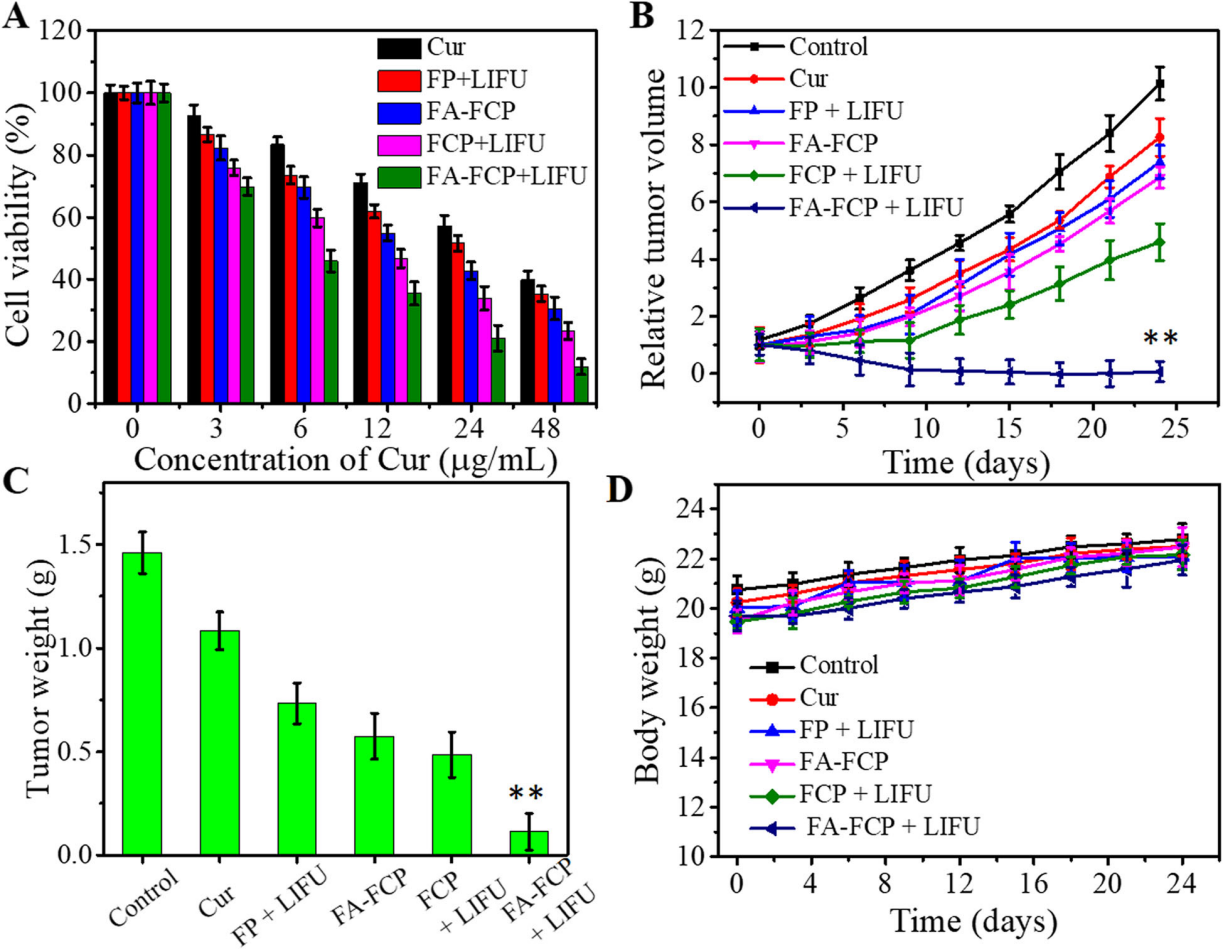
**a** Cell viability of SK-OV-3 cells incubated with different concentration of Cur, FP + LIFU, FA-FCP, FCP + LIFU, and FA-FCP + LIFU. **b** Relative tumor volume of tumor-bearing nude mice in control, Cur, FP + LIFU, FA-FCP, FCP + LIFU, and FA-FCP + LIFU groups, respectively. **p < 0.01, compared with the other groups. **c** Post-treatment tumor weight of tumor-bearing nude mice in control, Cur, FP + LIFU, FA-FCP, FCP + LIFU, and FA-FCP + LIFU groups, respectively. **p < 0.01, compared with the other groups. **d** Body weight of tumor-bearing nude mice in control, Cur, FP + LIFU, FA-FCP, FCP + LIFU, and FA-FCP + LIFU groups, respectively.

In vitro cytotoxicity of FA-FCP was investigated by CCK-8 assay. As shown in Fig. 7a, the cell viability of all the groups displayed a Cur concentration-dependent pattern. Without LIFU, compared with free Cur, FA-FCP showed higher inhibition in cell viability at all the concentrations, possibly due to the FA targeting effect. However, the cell viability inhibition of FA-FCP + LIFU was significantly higher than that of FP + LIFU, FCP + LIFU and FA-FCP without LIFU, indicating that FA-FCP combined with LIFU irradiation offered better anti-tumor efficiency. This result was mainly due to the presence of LIFU and the acid environment of the lysosome, both of which could induce the drug release and cavitation effect, and the FA-mediated tumor cells target effect [36–38].

In vivo anticancer effect of the FA-FCP was further investigated with tumor-bearing nude mice. According to the tumor accumulation result of FA-FCP, LIFU was conducted 18 h after intravenous injection of samples every 2 days for three times in total from day one. As shown in Fig. 7b and c, the relative tumor volume in the FA-FCP + LIFU group significantly decreased, compared with the control, Cur, FA-FCP, FP + LIFU and FCP + LIFU groups after 24 days of treatment. The tumor weight in the FA-FCP and FCP + LIFU groups was significantly less than that in the control and Cur groups, while it was even more inhibited in the FA-FCP + LIFU group. During the treatment, there had been no significant loss in body weight in these groups (Fig. 7d). The brilliant anti-tumor effect of FA-FCP combined with LIFU was mainly due to the targeting aggregation of FA-FCP in tumor and acoustics/pH-responsive drug-release ability [39–41]. Additionally, this enhanced anti-tumor therapeutic efficacy of the FA-FCP + LIFU might be explained by the delayed clearance of the nanoparticles at the tumor site due to the prolonged half-life of FA-FCP in the bloodstream [42].

### In Vitro and In Vivo Biocompatibility

**Fig. 8:**
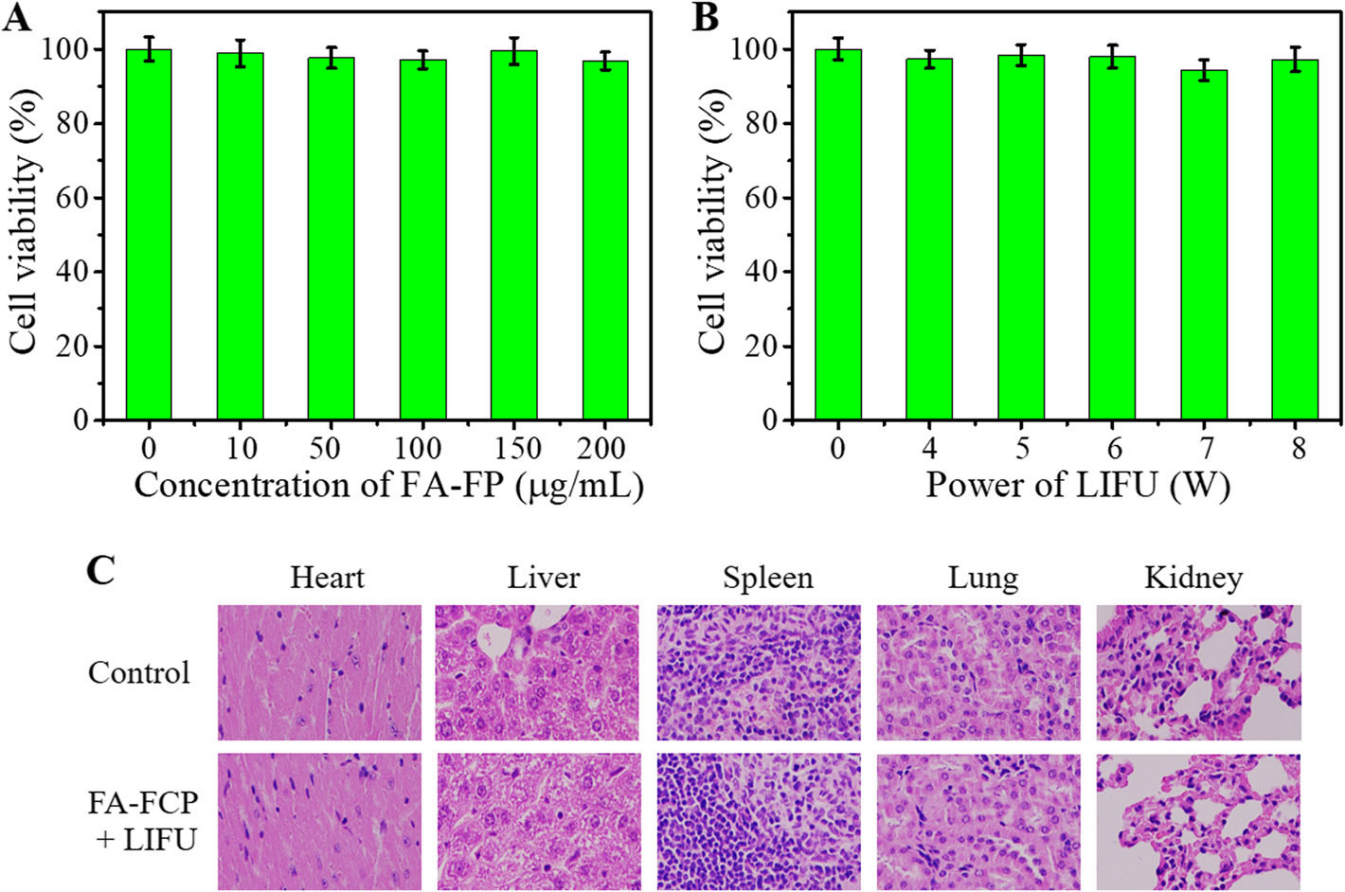
**a** Cell viability of SK-OV-3 cells after incubated with FA-FP for 24 h. **b** Cell viability of SK-OV-3 cells after treatment by different power of LIFU. **c** H&E staining of major organs of control group and FA-FCP group at 24 days after being intravenously injected with nanoparticles (×200)

In vitro and in vivo biocompatibilities of FA-FCP were evaluated by CCK-8 assay and H&E staining analysis. As shown in Fig. 8a and b, the cell viability of the drug-carrying FA-FP and power of LIFU from 0 to 8 W were all > 90%, indicating no significant cytotoxicity of the FA-FP and the power of LIFU used in this work in vitro. Figure 8c shows the H&E staining images of major organs of the mice treated with FA-FCP + LIFU, displaying no histological changes compared with the control group. These results demonstrated the high biocompatibility of FA-FCP in vitro and in vivo.

## Conclusion

In summary, we prepared a multifunctional FA-FCP loaded with the anticancer drug Cur and tumor-targeting molecule FA, using ferritin nanocages, which resulted in contrast-enhanced US imaging ability and precise targeting in chemotherapy procedures. The newly synthesized FA-FCP showed high in vitro and in vivo biocompatibility. In addition, FA-FCP exhibited superb tumor targeting ability, acoustic/pH-triggered Cur release, and acoustic-responsive phase transition by LIFU for ultrasound imaging. Given these unique properties of FA-FCP, it can be applied as a significant tumor inhibition factor with no systemic toxicity. It is expected that such a novel and biocompatible theranostic nanoplatform will integrate ultrasound imaging with improved therapeutic efficacy to provide a promising paradigm for the treatment of cancer.

## Supplementary information

**Additional file 1: Figure S1.** The AFM image of FA-FCP. **Figure S2.** The fluorescence intensity of FITC labelled FCP and FA-FCP at the same concentration.

## Data Availability

The conclusions made in this manuscript are based on the data (main text and figures) presented and shown in this paper

## Abbreviations

US: Ultrasound
FA: Folic acid
FRT: Ferritin
PFH: Perfluorohexane
LIFU: Low-intensity focused ultrasound
ADV: Acoustic droplet vaporization
FITC: Fluorescein isothiocyanate
CCK-8: Cell Counting Kit-8
FBS: Fetal bovine serum
EDC: 1-ethyl-3-(3-dimethylaminopropyl) carbodiimide
NHS: *N*-hydroxysuccinimide
Cur: Curcumin
