# Supplementary material for: Curcumin-Loaded Nanoparticles with Low-Intensity Focused Ultrasound-Induced Phase Transformation as Tumor-Targeted and pH-Sensitive Theranostic Nanoplatform of Ovarian Cancer
Source: Nanoscale Res Lett. 2020 Apr 7;15:73. doi: 10.1186/s11671-020-03302-3 (PMC7138896; doi:10.1186/s11671-020-03302-3)
Supplement: Supplementary file 1 — Additional file 1: Figure S1. The AFM image of FA-FCP. Figure S2. The fluorescence intensity of FITC labelled FCP and FA-FCP at the same concentration. [file 11671_2020_3302_MOESM1_ESM.docx]

**Supplementary Data**

**Curcumin-loaded nanoparticles with low-intensity focused ultrasound-induced phase transformation as tumor-targeted and pH-sensitive theranostic nanoplatform of ovarian cancer**

Xiaoxia Guo^1^, Jie Mei^1^, Yong Jing^2^, Shiguang Wang^2^*

^1^ Department of Obstetrics and Gynecology, Sichuan Academy of Medical Sciences & Sichuan Provincial People’s Hospital, Chengdu, Sichuan 610041, China

^2^ Department of Imaging, Eastern Hospital of Sichuan Academy of Medical Sciences & Sichuan Provincial People’s Hospital, Chengdu, Sichuan 610000, China

*Corresponding author: Shiguang Wang

Email: haocg_m@163.com, wangsg_im@hotmail.com

Telephone: + 86-13684006886

Address: No. 585 Honghe North Road, Longquanyi District, Chengdu, Sichuan 610000, China


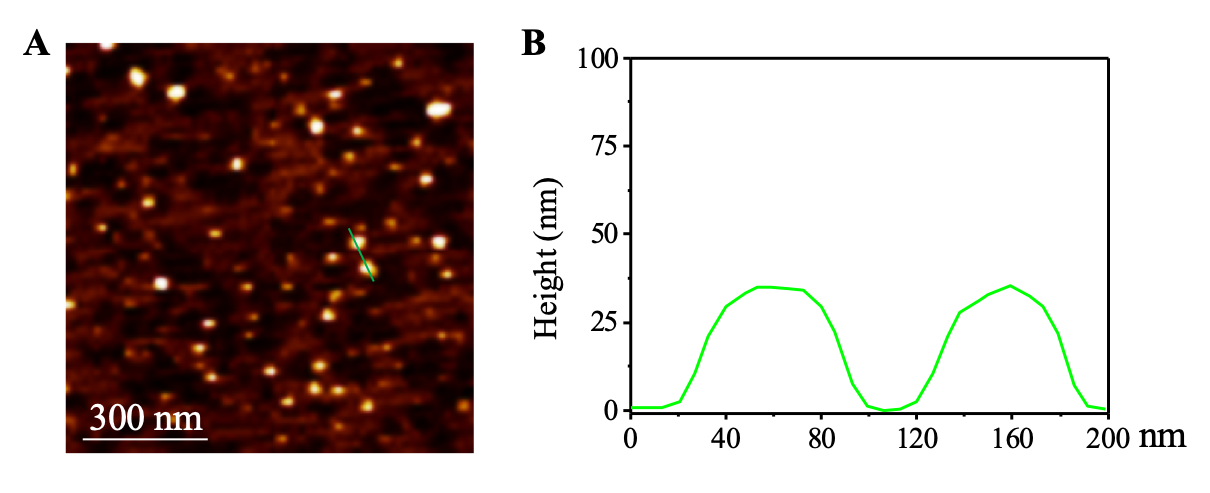


Figure S1. The AFM image of FA-FCP.





**Figure S2.** The fluorescence intensity of FITC labelled FCP and FA-FCP at the same concentration.
